# The Host Protein Calprotectin Modulates the *Helicobacter pylori cag* Type IV Secretion System via Zinc Sequestration

**DOI:** 10.1371/journal.ppat.1004450

**Published:** 2014-10-16

**Authors:** Jennifer A. Gaddy, Jana N. Radin, John T. Loh, M. Blanca Piazuelo, Thomas E. Kehl-Fie, Alberto G. Delgado, Florin T. Ilca, Richard M. Peek, Timothy L. Cover, Walter J. Chazin, Eric P. Skaar, Holly M. Scott Algood

**Affiliations:** 1 Veterans Affairs Tennessee Valley Healthcare Services, Nashville, Tennessee, United States of America; 2 Department of Medicine, Vanderbilt University School of Medicine, Nashville, Tennessee, United States of America; 3 Department of Pathology, Microbiology and Immunology, Vanderbilt University School of Medicine, Nashville, Tennessee, United States of America; 4 Department of Biochemistry, Vanderbilt University School of Medicine, Nashville, Tennessee, United States of America; 5 Center for Structural Biology, Vanderbilt University School of Medicine, Nashville, Tennessee, United States of America; Fred Hutchinson Cancer Research Center, United States of America

## Abstract

Transition metals are necessary for all forms of life including microorganisms, evidenced by the fact that 30% of all proteins are predicted to interact with a metal cofactor. Through a process termed nutritional immunity, the host actively sequesters essential nutrient metals away from invading pathogenic bacteria. Neutrophils participate in this process by producing several metal chelating proteins, including lactoferrin and calprotectin (CP). As neutrophils are an important component of the inflammatory response directed against the bacterium *Helicobacter pylori*, a major risk factor for gastric cancer, it was hypothesized that CP plays a role in the host response to *H. pylori*. Utilizing a murine model of *H. pylori* infection and gastric epithelial cell co-cultures, the role CP plays in modifying *H. pylori* -host interactions and the function of the *cag* Type IV Secretion System (*cag* T4SS) was investigated. This study indicates elevated gastric levels of CP are associated with the infiltration of neutrophils to the *H. pylori*-infected tissue. When infected with an *H. pylori* strain harboring a functional *cag* T4SS, calprotectin-deficient mice exhibited decreased bacterial burdens and a trend toward increased *cag* T4SS -dependent inflammation compared to wild-type mice. *In vitro* data demonstrate that culturing *H. pylori* with sub-inhibitory doses of CP reduces the activity of the *cag* T4SS and the biogenesis of *cag* T4SS-associated pili in a zinc-dependent fashion. Taken together, these data indicate that zinc homeostasis plays a role in regulating the proinflammatory activity of the *cag* T4SS.

## Author Summary

*Helicobacter pylori* is a bacterium that colonizes the stomach and causes gastric diseases. Some strains of *H. pylori* possess a secretion system that has the capacity to inject a cancer-causing protein into host cells. The activity of this secretion system contributes to the development of inflammation and is linked to the development of gastric cancer. Here, we show that the host protein calprotectin, which has the ability to bind and sequester nutrient metals from invading pathogens, can directly repress *H. pylori* secretory activity and the production of secretion-associated pili in a zinc-dependent manner. *H. pylori*-infected animals lacking calprotectin trend toward having more gastric inflammation and a significantly lower bacterial burden than infected animals that express calprotectin; these differences are not observed when animals are infected with a strain of *H. pylori* that lacks an active secretion system. Thus, a better understanding of how nutritional immunity modulates this secretion system could help us develop novel antimicrobial therapeutic strategies targeting secretory processes in *H. pylori*.

## Introduction

*Helicobacter pylori* is a Gram-negative bacterial pathogen that colonizes half of the world's population and contributes to a variety of disease outcomes, including peptic or duodenal ulcer disease, gastric adenocarcinoma, and mucosal-associated lymphoid tissue (MALT) lymphoma [1]. The most common manifestation of *H. pylori*-related disease is chronic gastric inflammation (non-atrophic chronic gastritis), which can potentially advance to multifocal atrophic gastritis, a precancerous lesion [2]. *H. pylori*-induced gastritis is characterized by neutrophil and mononuclear leukocyte infiltration to the lamina propria, involving cells in both the innate and adaptive arms of the immune response.

This gastric mucosal inflammatory response to *H. pylori* is enhanced if persons are infected with strains that possess a *cag*- type IV secretion system (*cag* T4SS). The *cag* T4SS is a macromolecular assembly that is responsible for translocating the oncogenic effector molecule, CagA and peptidoglycan, into host cells [3], [4]. These translocated effectors elicit a variety of host cell responses, including activation of nuclear factor κB (NFκB) and secretion of the proinflammatory cytokines, IL-1β and IL-8 [5]–[8]. The latter is associated with recruitment of innate immune cells, including neutrophils [9]. Neutrophil recruitment to the gastric mucosa is also enhanced by Th17 and Th1 responses.

Neutrophils are capable of controlling microbial infections via phagocytosis and subsequent production of reactive oxygen and reactive nitrogen intermediates, neutrophil extracellular trap (NET) formation, and production of antimicrobial factors [10], [11]. One such example is calprotectin (CP) [12]. CP, a heterodimer of S100A8 and S100A9 subunits (also known as Mrp8/14, calgranulin A/B, cystic fibrosis antigen). CP comprises about 50% of the neutrophil's cytoplasmic protein content and is a critical component of the host nutrient withholding process termed nutritional immunity [13], [14]. Humans and other mammals restrict access to essential metals through this nutritional immunity mechanism as a means to prevent infection with pathogenic organisms.

CP binds manganese and zinc with high affinity, effectively starving bacteria of these essential nutrients. There are two transition metal binding sites in CP; site 1 (S1; 6 His site) binds manganese and zinc, and site 2 (S2; 3 His Asp site) binds zinc only [15], [16]. Mutagenesis of CP's two metal binding sites has produced a site 1 mutant (ΔS1) that binds zinc only, a site 2 mutant (ΔS2) capable of binding both manganese and zinc, and a double site mutant (DS CP) incapable of binding manganese and zinc. Previous reports have indicated that CP exhibits antimicrobial activity against numerous microorganisms, including *Salmonella enterica* serovar Typhimurium, *Staphylococcus aureus*, *Escherichia coli*, *Borrelia burgdorferi*, *Listeria monocytogenes*, *Candida albicans*, *Acinetobacter baumannii*, *Staphylococcus epidermidis*, *Staphylococcus lugdunensis*, *Enterococcus faecalis*, *Pseudomonas aeruginosa*, and *Shigella flexneri* [12], [14], [15], [17]–[22]. CP has been demonstrated to enhance pathogenic *Salmonella* persistence in the inflamed gut [18] as well as increase neutrophil killing of *S. aureus* [14]. Concentrations of calprotectin in the tissue have been reported to be as high as 20 mg/ml in response to bacterial infections [23]. Expression of CP subunits S100A8 and S100A9 in inflamed gastric tissues of *H. pylori*-infected persons has been reported in the literature [24]. However, the interaction between CP and *H. pylori* has not been previously investigated.

*H. pylori*-associated inflammation is dynamic and recently published results indicate that *H. pylori* can modulate its *cag* T4SS activity in response to inflammation [25]. Here, we report a study to determine the role of CP in control of *H. pylori* colonization and pathogenesis. We find that gastric CP levels are elevated in *H. pylori*-infected humans and rodents, and that most of the CP localizes to neutrophils in *H. pylori*-infected tissues. We also demonstrate that *H. pylori*-infected CP-deficient mice (A9−/− mice) have decreased bacterial burden and a trend towards increased gastric inflammation compared to infected WT mice, a phenotype which is not observed in mice infected with an *H. pylori* isogenic *cagE* mutant, which lacks *cag* T4SS activity. Finally, we show that CP represses *cag* T4SS activity and *cag* T4SS-associated pilus production via zinc sequestration.

## Results

### Calprotectin is elevated in *H. pylori*-infected mice and humans

**Figure 1:**
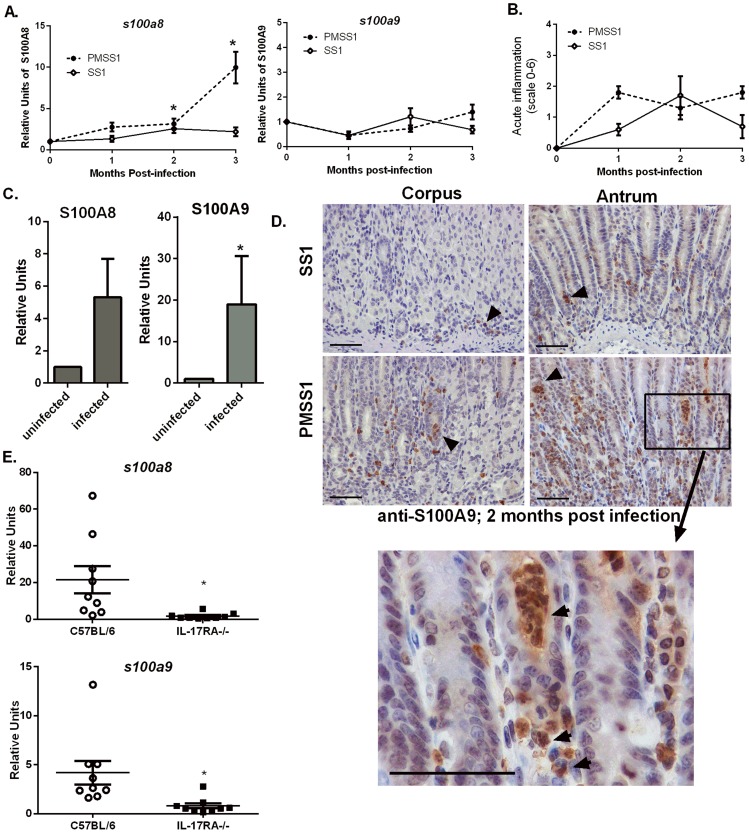
Host CP (S100A8/A9) is elevated in *H. pylori* infected stomach tissue. A) *s100a8/s100a9* transcript abundance in RNA extracted from C57BL/6 mice infected with *H. pylori* PMSS1 or SS1 for 1, 2, or 3 months relative to uninfected animals as determined by real-time RT-PCR analysis. Points indicate mean relative units of transcript abundance +/− SEM (levels of *s100a8* in PMSS1-infected mice compared to uninfected mice; 1 mo *p* = 0.0511; 2 mo *p* = 0.0432; 3 mo *p* = 0.0127; levels of *s100a8* in SS1-infected mice compared to uninfected mice at 2 mo *p* = 0.0623 Student's *t* test). (B) Inflammation scores of *H. pylori* infected mice at 1, 2, and 3 months post infection. (C) *s100a8/s100a9* transcript abundance in RNA extracted from gastric biopsies derived from human patients, which were either *H. pylori*-positive or *H. pylori*-negative (*s100a8 p* = 0.15; *s100a9* *p = 0.05). Bars indicate mean relative units of transcript abundance +/− SEM. Each experimental group represents≥5 individuals (mice or human samples). D) Gastric samples derived from *H. pylori* PMSS1-infected WT mice or SS1-infected WT mice at 2 months post-infection were analyzed via immunohistochemistry using a polyclonal antibody to S100A9 (scale bars are 50 microns). E) Real-time RT-PCR was performed on gastric tissue to quantify *s100a8* and *s100a9* transcript abundance from WT (C57BL/6 mice) and IL-17RA-/- mice infected with PMSS1. Data represent relative units of transcript abundance +/− SEM in WT mice and IL-17RA-/- mice, *p = 0.0169 and *p* = 0.0143, respectively.

In order to determine if CP is elevated in the context of *H. pylori* infection, real-time RT-PCR analysis of *s100a8* and *s100a9* transcripts in RNA isolated from either mouse or human gastric tissue was performed. For the first mouse study, RNA was isolated from gastric tissue of mice that had been infected with *H. pylori* PMSS1 or SS1 for 1, 2, and 3 months and *s100a8* and *s100a9* expression was compared to that of uninfected mice. CP subunit *s100a8* was significantly increased in gastric tissue in response to *H. pylori* infection (Figure 1A). Transcript levels of CP subunit *s100a9* did not significantly increase, but it has been demonstrated that the S100A9 protein is stabilized by increased S100A8 expression [18]. Corresponding inflammation scores are presented for these infections (Figure 1B). In a second study, human gastric biopsy samples were divided into *H. pylori*-negative samples and *H. pylori*-positive samples. Gene expression of both *s100a8* and *s100a9* subunits were elevated in *H. pylori*-infected biopsy samples compared to *H. pylori*-negative samples (Figure 1C).

### CP expression in the stomach localizes to neutrophils

CP was elevated in response to *H. pylori* infection in both human and murine stomach tissues, a phenomenon we hypothesize is driven by the increased presence of neutrophils. An immunohistochemical staining approach was used to evaluate the localization of CP within *H. pylori* PMSS1 or SS1-infected murine stomach tissues. Microscopy analyses revealed that the majority of CP is localized in association with neutrophils within the gastric tissue (Figure 1D).

Since immunohistochemistry staining revealed that CP was mainly localized in proximity to host neutrophils (Figure 1D) and since CP comprises 40–60% of the total protein in the neutrophil cytoplasm [26], [27], we hypothesized that recruitment of neutrophils to the *H. pylori*-infected stomachs correlates with increases in CP expression. To test this hypothesis, IL-17 receptor A-deficient mice (IL-17RA-/-) were used. Previously published data indicate that these mice have a defect in IL-17 signaling, a prerequisite for the maintenance of neutrophil recruitment to the stomach during chronic *H. pylori* infection [28]. At 3 months post-infection, *H. pylori* infected IL-17RA-/- mice exhibit significantly decreased PMN infiltration compared to *H. pylori* infected WT mice [28]. Thus, the 3 month time point was chosen for these analyses. Real-time RT-PCR analysis of CP subunit expression revealed that PMSS1-infected IL-17RA-/- mice have diminished *s100a8* and *s100a9* expression compared to PMSS1-infected wild-type (WT) animals (Figure 1E), demonstrating that increased abundance of CP was correlated to the presence of a neutrophilic infiltrate.

### CP affects growth and viability of *H. pylori*

**Figure 2:**
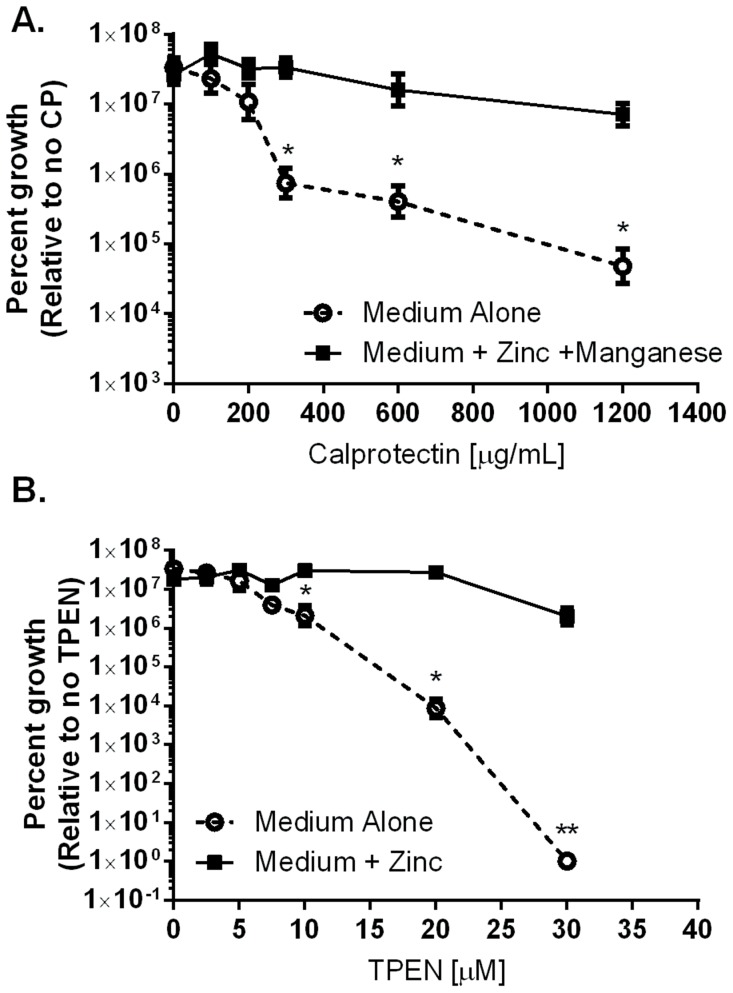
Inhibition of *H. pylori* growth *in vitro* by CP or TPEN is dose dependent. A) WT *H. pylori* were cultured for 24 hours in medium alone or medium supplemented with 50 µM zinc chloride plus 50 µM manganese chloride (Medium+Zinc+Manganese) and with increasing concentrations of CP. B) WT *H. pylori* were cultured for 24 hours in medium alone or medium supplemented with 100 µM zinc chloride (Medium+Zinc) and with increasing concentrations of TPEN, a synthetic zinc chelator. Bacterial growth was evaluated by spectrophotometric OD_600_ reading. The percent growth was determined by comparing the OD_600_ reading of each culture to controls grown in medium alone. *p<0.05, **p<0.01, ***p<0.001, Student's *t*-test, n = 3 biological replicates.

CP has been demonstrated to inhibit bacterial growth via sequestration of nutrient manganese and zinc [16]–[18]. We hypothesized that CP is elevated in response to *H. pylori* infection as part of a host strategy to inhibit bacterial proliferation within the gastric niche. To test this proposal, *in vitro* growth assays were performed in modified bacteriological medium. Analysis of bacterial growth curves (OD_600_) and colony forming units (CFU/mL) revealed that wild-type CP (CP) at 300 µg/mL significantly inhibited *H. pylori* growth (Figure 2 and Figure S1). The addition of exogenous manganese and zinc (50 µM of zinc chloride and 50 µM manganese chloride) restored growth to control levels. In addition to investigating the ability of CP to inhibit growth of *H. pylori*, the effects of three previously generated mutants of CP's metal binding sites (DS CP, ΔS1, and ΔS2) were investigated [15]. The DS CP harboring inactivation of both S1 (manganese and zinc binding) and S2 (zinc binding alone) sites was unable to inhibit bacterial growth (Table S1). The ΔS1 mutant at 1200 µg/mL was able to inhibit bacterial growth, as was the ΔS2 mutant (Table S1). These results indicate that CP inhibited *H. pylori* growth *in vitro* at concentrations above 300 µg/mL, and that the antibacterial activity is dependent on CP's ability to sequester metal.

### CP affects *H. pylori* colonization and *H. pylori*-induced inflammation in a mouse model

**Figure 3:**
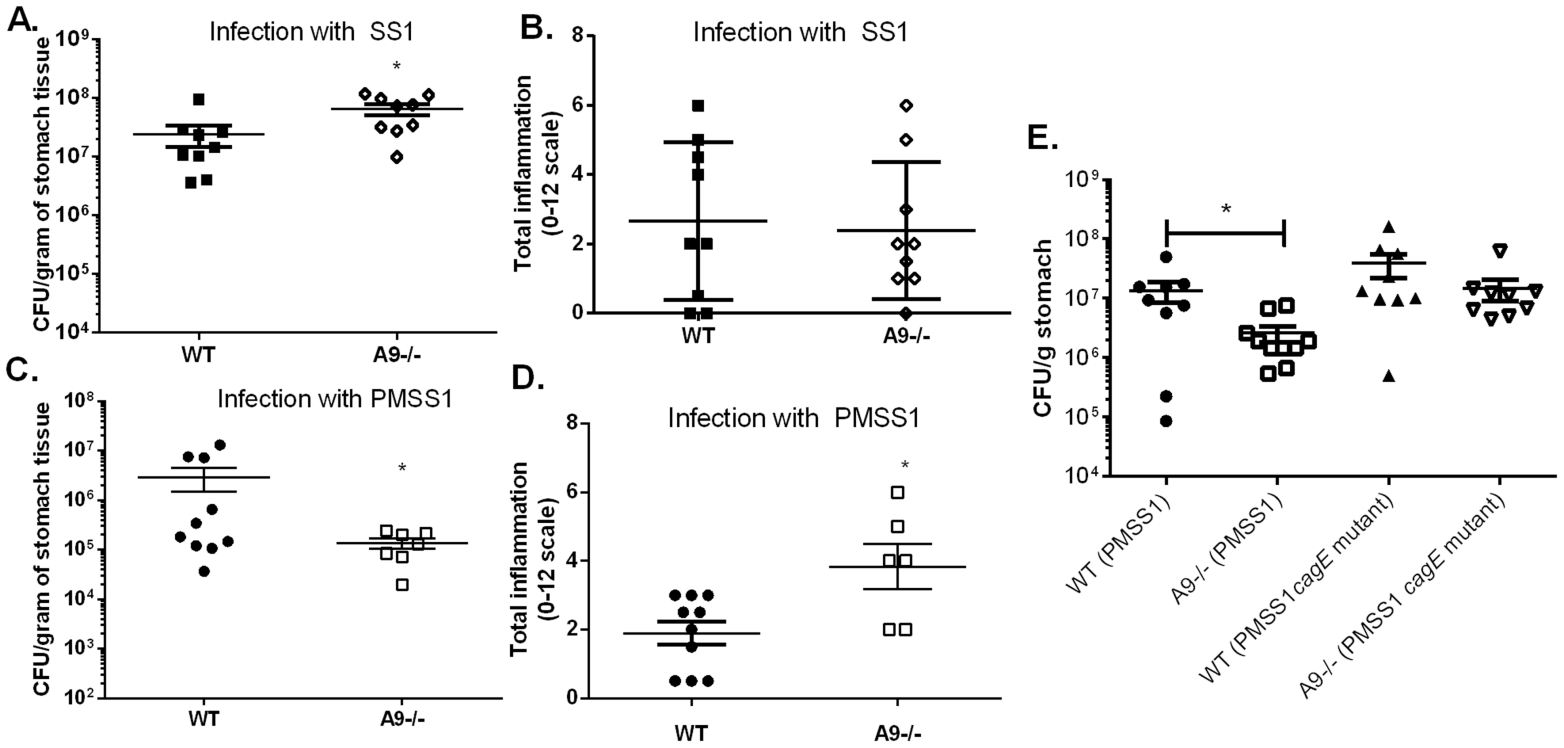
The presence of calprotectin increases bacterial burden and reduces inflammation in WT mice in a *cag* T4SS-dependent manner. WT mice and calprotectin-deficient mice (A9-/-) were infected with either WT *H. pylori* strain SS1, PMSS1 or an isogenic mutant with an inactivation of the *cagE* locus, and sacrificed 6 weeks post-infection. A) Bacterial burden was higher in A9-/- mice compared to WT mice in groups infected with WT *H. pylori* SS1 strain. B) There is no significant difference in inflammation in A9-/- mice compared to WT mice in *H. pylori* SS1 infected mice. C) Bacterial burden was lower in A9-/- mice compared to WT mice in groups infected with WT *H. pylori* PMSS1 strain (*p* = 0.0325). D) Histological analyses of inflammation demonstrate that PMSS1 infected A9-/- mice had significantly higher inflammation than PMSS1 infected WT mice. Statistical analyses were performed for inflammation scores using Mann Whitney U test. E) Bacterial burden was diminished in A9-/- mice compared to WT mice in groups infected with WT *H. pylori* PMSS1 strain (*p* = 0.038), but not in groups infected with the PMSS1 *cagE* mutant (*p* = 0.4699). Data represent the mean +/− SEM per each group (n = 8–9 animals per group). Statistical analyses were performed for bacterial burden using Student's *t* test. *p<0.05.

Because CP can inhibit the growth of *H. pylori in vitro*, we hypothesized that this host protein would also contribute to control of the bacterial burden *in vivo*. To test this hypothesis, both WT and CP-deficient (A9-/-) mice were orogastrically infected with *H. pylori* strain SS1 or PMSS1; the former strain lacks a functional *cag* T4SS, while the latter expresses a functional *cag* T4SS. In analysis of animals at 6 weeks post-infection, A9-/- mice infected with the SS1 strain had significantly higher levels of *H. pylori* compared to WT mice infected with the SS1 strain (Figure 3A, *p* = 0.014), A9-/- mice infected with PMSS1 had significantly fewer CFU per gram of tissue compared to PMSS1-infected WT mice (Figure 3C, *p* = 0.0325). Bacterial burden and inflammation have been shown to have a reciprocal relationship in *H. pylori* models of murine infection [29]–[31]. This suggests that CP-deficient mice may have an increase in the inflammatory response to *H. pylori*. To test this hypothesis, inflammation in WT and A9-/- mice was evaluated via histological analysis and scoring. The A9-/- mice infected with SS1 did not have significant differences in inflammation compared to WT mice (Figure 3B), but A9-/- mice infected with PMSS1 had significantly higher inflammation scores than WT-infected animals (*p* = 0.04; Figure 3D). These results suggested that the absence of CP results in increased gastric inflammation during *H. pylori* infection with a strain expressing a functional *cag* T4SS (the SS1 strain lacks a functional *cag* T4SS), which may explain the decreased bacterial burden. We also investigated the effect of CP on chronic infection in the mice at later time points, up to 3 months post infection. At this timepoint, there were no significant differences in the colonization in the *H. pylori*-infected A9-/- mice compared to WT mice, but there was a trend toward increased inflammation in *H. pylori*-infected A9-/- mice compared to *H. pylori*-infected WT mice (SS1 infection *p* = 0.06; PMSS1 infection *p* = 0.13). We observed that the stomachs of A9-/- mice became infected with fungus by 3 months, which complicates the interpretation of results at this timepoint.

We hypothesized that CP modulates the *cag* T4SS since the activity of this virulence factor is associated with *H. pylori*-induced inflammation. To test this hypothesis, WT and A9-/- mice were infected with either PMSS1 or an isogenic *H. pylori* PMSS1 *cagE* mutant. Since CagE is the ATPase that powers *cag* T4SS assembly, *H. pylori* strains deficient for CagE do not have a functional *cag* T4SS and do not form *cag* T4SS pili [32]. Again, when infected with the PMSS1 strain, the bacterial burden was significantly lower (Figure 3E) and there was a trend toward higher inflammation in the A9-/- infected mice compared to the WT mice (Figure S2A). When infected with the PMSS1 *cagE* mutant, there was no significant difference in bacterial burden (Figure 3E) or inflammation (Figure S2) in A9-/- mice compared to WT mice at 6 weeks post-infection. To further quantify the gastritis in these mice, flow cytometry was performed at 6 weeks post-infection. There was a trend toward greater numbers of gastric PMNs and monocytes in PMSS1-infected A9-/- mice compared to PMSS1-infected WT mice (*p* = 0.057 and *p* = 0.097, respectively; Figure S2B, C). Assessment of CP levels (transcript levels of *s100a8* and s*100a9*) by realtime rtPCR showed no difference in expression when comparing *H. pylori* infected WT mice infected with PMSS1 or the PMSS1 *cagE* mutant (Table S2). Thus differences in bacterial burden and inflammation between PMSS1-infected WT and the A9-/- mice are likely *cag* T4SS-dependent (Figure 3E and Figure S2). These data suggest that CP may repress the activity of the *cag* T4SS *in vivo*.

### CP inhibits activity of the *H. pylori cag* T4SS

**Figure 4:**
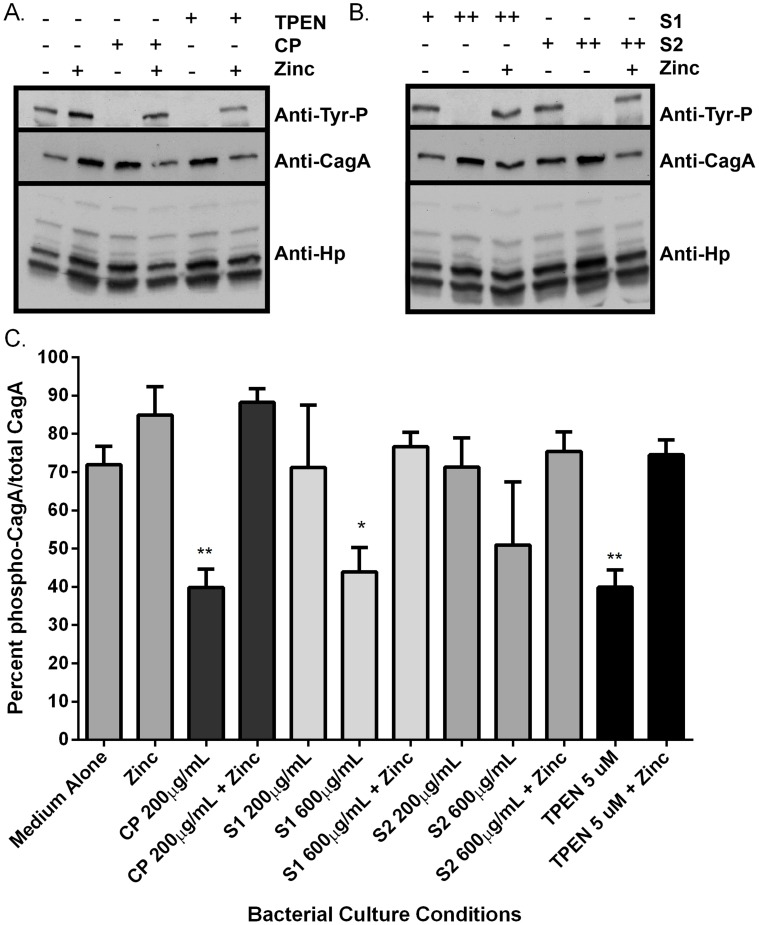
Calprotectin inhibits CagA translocation into gastric epithelial cells. Analysis of CagA phosphorylation upon translocation into AGS cells and total CagA. A) Prior to co-culture with AGS cells, bacteria were grown in medium alone or medium supplemented with various additives (100 µM zinc chloride, or with CP (200 µg/mL), or the synthetic zinc chelator TPEN, in the presence or absence of 100 µM zinc chloride). B) Bacteria were grown in the presence of mutant forms of CP that lack S1 or S2 at 200 µg/mL (+) or 600 µg/mL (++) alone or in the presence of 100 µM zinc chloride prior to co-culture with AGS cells. C) Percentage total CagA that is phosphorylated was quantified by densitometry on immunoblots (p-TYR CagA/total CagA×100). Immunoblotting with anti-*H. pylori* was also performed as a loading control, as described previously [58]. Conditions of zinc sequestration repressed the CagA translocation activity of the T4SS. Supplementation with 100 µM zinc chloride (zinc) restored CagA phosphorylation to levels comparable to medium alone in all samples. Bars represent the mean +/− SEM per each group (n = 3–5 biological replicates). *p<0.05, **p<0.01, compared to medium alone (Student's *t* test).

**Figure 5:**
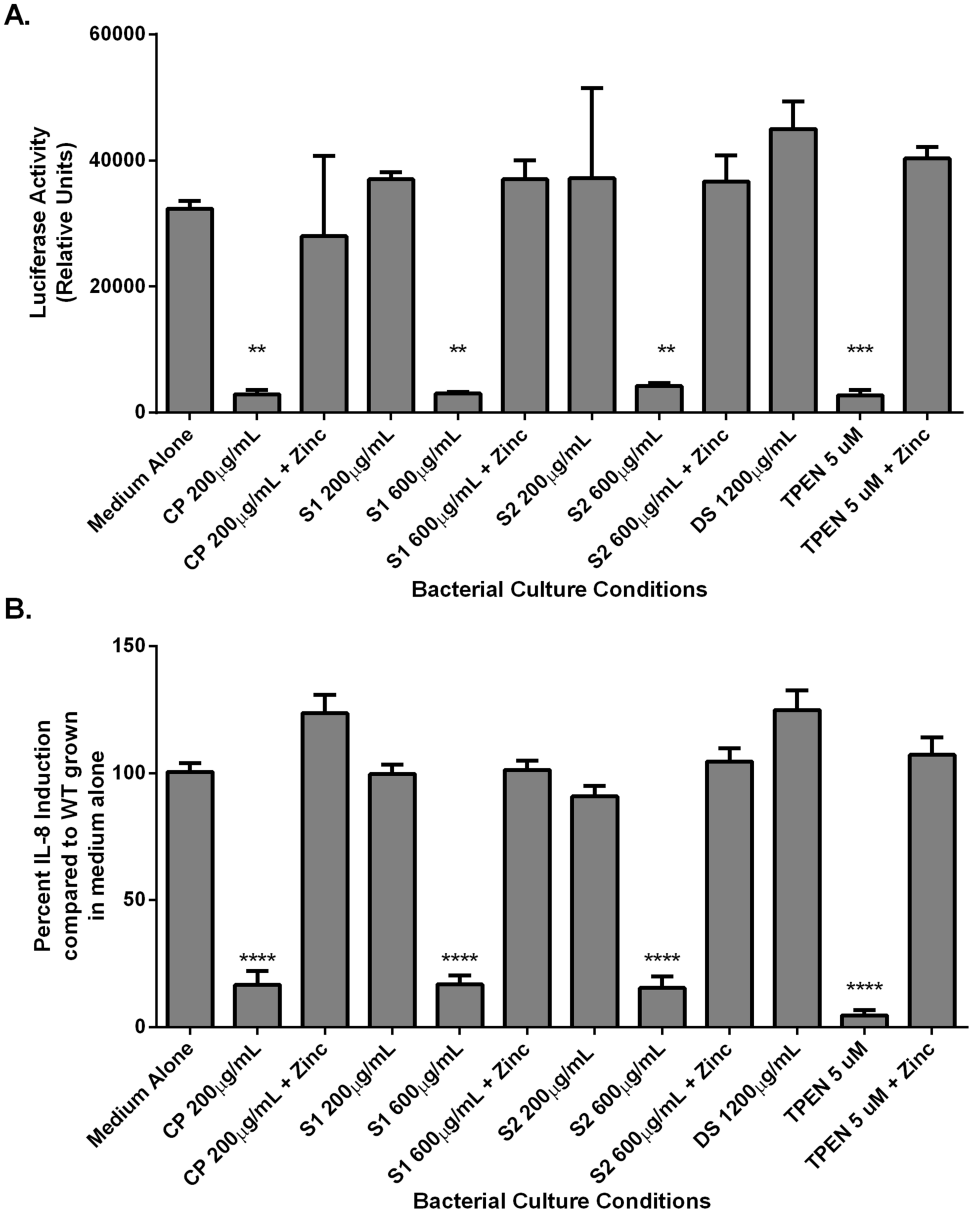
NFκB activation and IL-8 secretion in response to co-culture with *H. pylori* is dependent on zinc availability. Bacteria were grown in medium alone or medium supplemented with the synthetic zinc chelator TPEN (TPEN), wild-type CP (CP) at 200 µg/mL, or mutant forms of CP [ΔS1 or ΔS2 at 200 µg/mL or 600 µg/mL, or a double-site mutant (DS) at 1200 µg/mL] alone or in the presence of 100 µM zinc chloride (+Zinc) prior to co-culture with AGS cells. A) NFκB activation in human gastric epithelial cells co-cultured with *H. pylori* for 4 hours was quantified using a luciferase reporter assay. B) IL-8 secretion by human gastric epithelial cells was quantified using an IL-8 ELISA assay. Bars represent the mean +/− SEM per each group (n = 3–5 biological replicates). Asterisks indicate *p<0.05, ** *p*<0.01, *** *p*<0.001, **** *p*<0.0001, compared to medium alone (Student's *t* test).

A functional *cag* T4SS translocates the effector molecule, CagA, into host cells, where it is then phosphorylated [25]. Moreover, a functional *cag* T4SS is necessary for activation of NFκB (nuclear factor kappa-light-chain-enhancer of activated B cells) in human AGS gastric epithelial cells, which is a result of both CagA translocation and peptidoglycan recognition by NOD1 [33]. As a result of these cellular signaling events, IL-8 is produced and secreted by the AGS gastric epithelial cells. Therefore, the functional activity of the *cag* T4SS was measured with three assays; CagA translocation and phosphorylation (Figure 4), NFκB activation (Figure 5A), and IL-8 secretion by gastric epithelial cells (Figure 5B).

As an initial assessment of *cag* T4SS function, an assay for CagA translocation into AGS cells based on detecting phosphorylated CagA was performed. *H. pylori* were cultured in the presence or absence of CP for 4–6 hours prior to a 4 hour co-culture with AGS cells. Following washes to remove unbound bacteria, lysates of *H. pylori*-bound AGS cells were generated and separated by SDS-PAGE. The levels of phospho-CagA and total CagA in the extracts were determined by immunoblot analyses [25]. As shown in Figure 4A and C, reduced levels of phosphorylated CagA in *H. pylori*-AGS co-cultures were observed when *H. pylori* was pre-treated with sub-inhibitory (growth) doses of CP.

As mentioned earlier, activity of the *cag* T4SS leads to NFĸB activation and IL-8 secretion by gastric epithelial cells. To assess the ability of CP to inhibit cellular activation, *H. pylori* were cultured in the presence or absence of CP for 4–6 hours prior to a 1 hour co-culture with an AGS-NFĸB luciferase reporter cell line. As show in Figure 5, CP inhibits the ability of the *cag* T4SS to activate NFĸB (Figure 5A). To determine if CP also inhibits IL-8 secretion in this co-culture system, *H. pylori* were cultured in the presence or absence of CP for 4–6 hours prior to a 4 hour co-culture with AGS. IL-8 secretion was significantly lower in supernatants from AGS co-cultures with *H. pylori* pre-treated with CP compared to AGS co-cultures with untreated *H. pylori* (Figure 5B). To investigate whether these results were an effect of reduced adherence, a bacterial adherence assay was performed. There were no significant changes in *H. pylori* adherence as a consequence of treatment with either CP or TPEN alone or in the presence of an exogenous source of nutrient zinc (Figure S3).

To investigate which metal binding sites are critical for this phenotype and to determine if this phenotype is due to manganese sequestration or zinc sequestration, the same experiments were performed with CP ΔS1, ΔS2, and DS mutants. Mutagenesis of either the S1 site (manganese and zinc binding) or the S2 site (zinc binding alone) decreased the efficiency of CP's ability to repress the CagA translocation (Figure 4) and *cag* T4SS activity (Figure 5A and B). While 200 µg/ml of CP was sufficient to repress CagA translocation, NFkB activation and IL-8 production, due to a reduced ability to bind metal, higher levels (600 µg/ml) of ΔS1 and ΔS2 CP were needed to observe the same effects. The DS mutant, which does not bind any metals, was unable to ablate the *cag* T4SS-dependent NFĸB activation and had no effect on IL-8 induction (Figure 5A and B). Since the S1 mutant, which can only bind zinc, is still functional, nutrient zinc sequestration is responsible for the *cag* T4SS repression phenotype. The observation that CP is acting on the *cag* T4SS through a zinc sequestration-dependent pathway was consistent with experiments showing that the inhibitory effects of CP on both CagA phosphorylation and the activity of the *cag* T4SS were reversible with the addition of an exogenous zinc source (Figure 4 and 5).

To confirm that the effect of CP on the repression of *cag* T4SS was due to zinc chelation, a synthetic metal chelator which preferentially binds zinc, N,N,N′,N′-tetrakis (2-pyridylmethyl) ethylenediamine (TPEN), was tested for its ability to repress the *cag* T4SS. Bacteria pre-exposed to TPEN at concentrations below that necessary to inhibit growth (5 µM, Figures 2 and S1B) translocated less CagA than did untreated bacteria, as determined by immunoblot analysis for phospho-CagA (Figure 4A). Bacteria pre-exposed to TPEN caused less NFkB activation within the host cell (Figure 5A) and showed a diminished capacity to induce IL-8 secretion (Figure 5B). The addition of exogenous zinc to the *H. pylori* cultures reversed the phenotype, and the activity of the *cag* T4SS was restored (Figure 4 and 5). These data suggest that the activity of the *cag* T4SS is reduced by CP through a zinc-sequestration-dependent process.

### Metal sequestration by CP inhibits *cag* T4SS pilus biogenesis

**Figure 6:**
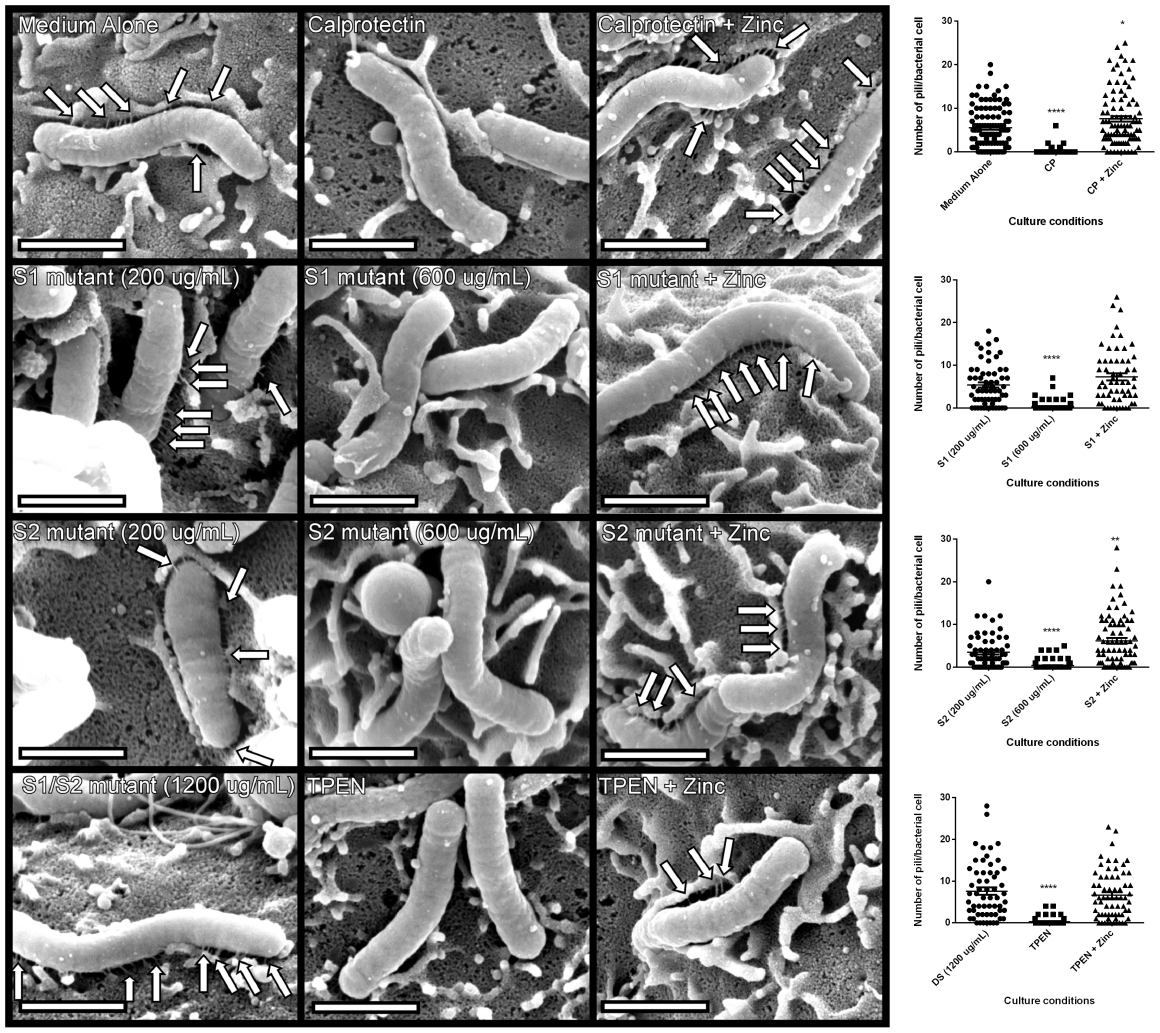
*H. pylori cag* T4SS pilus production is modulated by the zinc sequestration activity of calprotectin. High resolution FE-SEM analysis of *H. pylori* co-cultured with AGS human gastric epithelial cells. Bacteria were cultured in medium alone, or medium supplemented with wild-type CP (200 µg/mL) or mutant forms of CP that lack S1, S2 or both metal-binding sites (DS) at 200 µg/mL, 600 µg/mL, or 1200 µg/mL. Pilus formation was also examined using the synthetic zinc chelator, TPEN. Supplementation with 100 µM zinc chloride (+Zinc) restored T4SS pilus formation to levels comparable to medium alone in all samples. Arrows indicate *cag* T4SS pili formed at the host-pathogen interface. Magnification bars indicate 1 µm. Dot plot graphs indicate enumeration of pili per bacterial cell as quantified from representative micrographs derived from three biological replicates, and at least 20 fields from each replicate. Asterisks indicate *p<0.05, ** *p*<0.01, *** *p*<0.001, **** *p*<0.0001, compared to first condition on each graph (Student's *t* test).

Our co-culture experiments demonstrated that metal sequestration by CP leads to abrogated phosphorylation of CagA and inhibition of downstream cellular activation in gastric epithelial cells. We next tested the hypothesis that the observed inhibition of the *cag* T4SS-dependent phenotype was attributable to inhibition of *cag* T4SS pilus production. To test this, field emission gun-scanning electron microscopy (FEG-SEM) analysis of the bacterial-gastric epithelial cell (*H. pylori*-AGS cell) co-cultures was performed to visualize the *cag* T4SS pili, as previously described [25], [32], [34]. Briefly, bacteria were cultured for 4–6 hours prior to co-culture with AGS cells in the presence or absence of WT or mutant forms of CP or the synthetic zinc chelator, TPEN, alone or in the presence of an exogenous source of nutrient zinc. Bacteria were co-cultured with gastric cells in the absence of additives for 4 hours before samples were processed and analyzed by high resolution FEG-SEM. Pre-treatment of *H. pylori* with CP or TPEN reduced the number of *cag* T4SS pili visible at the host pathogen interface (Figure 6). The addition of exogenous zinc restored WT pili formation, suggesting that the zinc-chelation is responsible for this reduced pilus formation. Similarly, the ΔS1 and ΔS2 mutant CP proteins were unable to repress pilus formation at the same concentration as WT (200 µg/mL). However, when increasing the concentration of the ΔS1 and ΔS2 mutants to high concentrations (600 µg/ml), *cag* T4SS pilus formation was repressed. The higher concentration of both ΔS1 and ΔS2 mutant CP proteins was consistent with earlier results, where 600 µg/ml of these mutant proteins was necessary to observe decreased NFkB and IL-8 expression. Conversely, the DS mutant CP did not repress *cag* T4SS pilus formation, even at very high concentrations (1200 µg/ml). These data indicate that CP inhibits the production of *cag* T4SS-associated pili through zinc sequestration.

## Discussion

**Figure 7:**
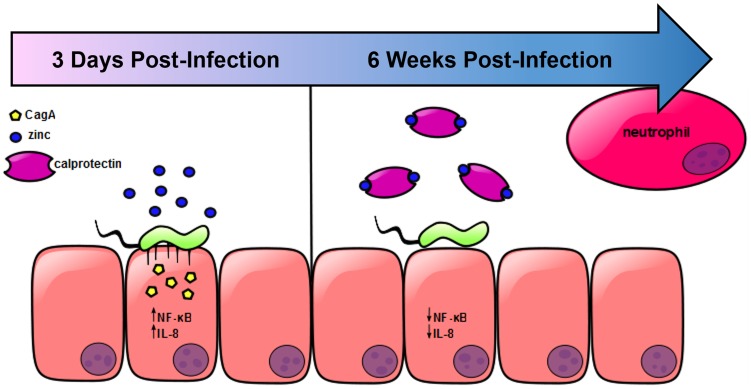
Model of *H. pylori cag* T4SS regulation in response to neutrophil recruitment and deposition of calprotectin at the site of infection. In the presence of available zinc, *H. pylori* elaborates a functional *cag* T4SS and translocates CagA into host epithelial cells, resulting in increased NFκB activation and IL-8 secretion. The chemokine IL-8 recruits neutrophils to the site of infection, increasing the amount of CP present. CP sequesters the nutrient zinc away from the bacterial cell, downregulating the activity of the *cag* T4SS, and thereby decreasing the translocation of CagA, NFκB activation and IL-8 secretion.

Results from these studies indicate that zinc homeostasis plays an important role in regulating the *cag* T4SS in *H. pylori*. Previous reports have shown that when *H. pylori* interacts with gastric epithelial cells, the *cag* T4SS translocates CagA into host cells leading to activation of c-Src and changes in the cytoskeleton [34]–[36]. A functional *cag* T4SS also increases the activation of NOD and NFkB [33], which ultimately results in the production of IL-8 and the recruitment of neutrophils [34]–[36]. Based on our results, we propose a model which is presented in Figure 7. In response to *H. pylori* infection, neutrophils are recruited to the site of infection. CP is deposited, and nutrient manganese and zinc are sequestered. Sequestration of zinc by CP represses *cag* T4SS pilus formation and CagA translocation, and results in diminished NFκB activation and IL-8 secretion. With reduced IL-8, less inflammation develops and the end result of CP-dependent zinc-sequestration is increased bacterial persistence.

*H. pylori* infection elicits a robust neutrophil response, resulting in increased expression of CP in the infected tissue, a result that agrees with previously published observations [24], [37]. In other models of infection, CP levels increase in response to bacterial infection [14], [18], [38], and levels vary from tissue to tissue [39]. It is likely that CP levels are dynamic within host gastric tissue during *H. pylori* infection because inflammation varies in a time- and location-dependent manner and is shaped by host genetics [2], [40], [41]. We hypothesized that CP is an important mediator of host-*H. pylori* interactions. Our results suggest that CP's activity against *H. pylori* may be dose-dependent. CP inhibits growth of *H. pylori* by sequestration of nutrient metals, an observation that agrees with the results seen with numerous other pathogens and supports the proposal that CP is part of the host response designed to restrict *H. pylori* burden *in vivo*. Yet when CP is absent, the bacterial burden is reduced by the increased inflammatory response. These data suggest that whether CP is present or absent, there is cross regulation between the bacteria and the host immune response, leading to a level of inflammation which controls bacterial burden but does not necessarily induce enough inflammation to completely clear the infection, and therefore, the bacteria persist.

In addition to indicating that CP restricts the growth of *H. pylori*, our work reveals that CP represses the activity of the *cag* T4SS, an important virulence factor that has been associated with carcinogenesis [42]. By utilizing CP mutants harboring inactivation of the metal binding sites within CP, our work demonstrated that both the site 1 and the site 2 mutant proteins have the capacity to inhibit the activity and biogenesis of pili associated with the *cag* T4SS. Since the CP mutant that can only bind zinc is still capable of inhibiting *cag* T4SS function and pilus biogenesis, this result proves that nutrient zinc sequestration is responsible for the *cag* T4SS repression phenotype, a result that is supported by restoration of *cag* T4SS activity and pilus biogenesis when an exogenous source of nutrient zinc is provided. At present, the components of the *cag* T4SS are not well defined and controversial [43], therefore it is not possible to determine whether pilus production is blocked at the level of pilus assembly, at the level of translation of pilus components or through some other mechanism. Interestingly, the *cag* T4SS has been reported to be induced by chelation of nutrient iron, a result that is reciprocal to the regulation imposed by sequestration of nutrient zinc, but still supports the contribution of micronutrients to the regulation of this important bacterial organelle [44]. There are other examples of reciprocal regulation of virulence factors by nutrient iron and zinc among bacterial pathogens. For example, in the gastrointestinal pathogen, enteropathogenic *E. coli* (EPEC), the expression and secretion of EPEC-associated secreted proteins (Esp) have been shown to be repressed by nutrient zinc and induced by nutrient iron [45].

Furthermore, CP-mediated metal sequestration has been associated with changes in bacterial virulence in other pathogens. For example, in the presence of CP, *S. aureus* superoxide defense is repressed, leading to diminished resistance to innate immune cells [16]. These previously published results reveal that CP can, in addition to repressing bacterial growth, also repress bacterial virulence to promote host defense strategies. In our murine model of infection, CP represses a major inflammation-promoting virulence factor, the *cag* T4SS. This activity may contribute to bacterial evasion of the immune system.

CP has been shown to be induced in the context of bacterial infection, and the utility of CP-deficient mice (A9 -/- mice) in these studies has proven to be important [14], [17], [18]. In a model of *S. aureus* infection, CP-deficient mice had an approximately 1-log increase in bacterial burden in their livers compared to WT mice [16]. Similarly, in a model of *A. baumannii* infection, CP-deficient mice exhibited a significant increase in bacterial burden relative to WT mice at 36 hours post-infection, but not at 72 hour post-infection, indicating that CP is important for controlling early infection, but other components of the host system can clear bacteria independently of CP [17]. Conversely, in a model of *S.* Typhimurium, *Salmonella* overcomes zinc sequestration by calprotectin to colonize the gut through production of the CznABC transporter. CznABC was also required to promote the growth of *S.* Typhimurium over that of competing commensal bacteria in the inflamed gut [18]. *H. pylori* bacterial burden is decreased in CP-deficient mice compared to WT mice, indicating that the activity of CP is not restricted to antimicrobial activity against *H. pylori*, but can also regulate bacterial virulence which may promote chronic colonization within the host gastric niche. In other words, CP repression of the *cag* T4SS could promote chronic colonization by reducing inflammation and increasing bacterial burdens.

In addition to repressing bacterial virulence, CP has other effects on bacterial pathogens. *S. aureus* upregulate two metal uptake systems, MntABC and MntH, to promote manganese acquisition in the presence of CP [39]. The metal-acquisition properties are important for staphylococcal resistance to CP as well, and mutants in *mntABC* or *mntH* have decreased growth compared to WT strains in increasing concentrations of CP [39]. Similarly, both *A. baumannii* and *S.* Typhimurium upregulate zinc acquisition machinery to resist the nutrient sequestration imposed by CP in inflamed tissues. Mutants in zinc acquisition in both of these pathogens have diminished capacity to compete with WT bacteria in rodent models of infection [17], [18]. Taken together, these data support a model in which zinc acquisition provides a selective advantage for invading pathogens in the presence of inflammation-associated antimicrobial peptides such as CP. It also supports the diverse roles that both manganese and zinc availability play in microbial responses within the vertebrate host.

The trace element zinc is essential for cell function, and zinc co-factors are prevalent in the bacterial proteome [46]. Less than 0.1% of zinc is found in serum plasma [47], indicating the vast majority of zinc is stored in the intracellular space, and thereby, unavailable to extracellular pathogens. Additionally, the immune system produces zinc-chelating molecules such as S100A8 and S100A9 that have the capacity to bind and sequester nutrient zinc from invaders [48]. Thus, zinc homeostasis is emerging as an important feature of the host-pathogen interaction. Zinc exposure has been associated with decreased adherence, biofilm formation and virulence factor expression in enteroaggregative *Escherichia coli* (EAEC) [49]. Additionally, macrophages have been shown to utilize intraphagosomal zinc accumulation as a strategy to poison bacterial pathogens such as *Mycobacterium tuberculosis* [50]. To combat this, *M. tuberculosis* elaborates heavy metal efflux P-type ATPases, metallothioneins, and a zinc exporter [50]. This strategy is not uncommon, as pathogens such as *Streptococcus pyogenes* and *Pseudomonas aeruginosa*, have been shown to express zinc efflux systems that are crucial for colonization of an animal host [51], [52]. Similarly, *H. pylori* encodes a novel metal efflux pump, CznABC, which is required for resistance to cadmium, nickel and zinc intoxication, as well as colonization of a vertebrate host [53]. These results implicate zinc as an important micronutrient signal at the host-pathogen interface that bacteria sense and respond to accordingly. *H. pylori* has likely evolved to sense the presence of zinc as well as the deprivation of zinc by CP to induce the appropriate cellular responses. Thus, CP is an important inflammation-associated environmental signal to the bacterial cell.

CP also has the ability to act as a damage-associated molecular pattern molecule (DAMP) within the host [54]. It has been demonstrated that CP activates toll-like receptor 4 (TLR4) and signaling through nuclear factor ĸB, which ultimately leads to increased inflammatory responses [54], [55]. Additionally, CP is hypothesized to interact with the receptor for advanced glycation end products (RAGE) to promote chronic inflammation [56]. In the context of our murine model of infection, CP itself could potentially contribute to inflammatory processes. However, in the presence of an *H. pylori* infection, the presence of CP is associated with diminished inflammation, which can be attributed to the zinc-dependent regulation of the proinflammatory *cag* T4SS.

In conclusion, we propose a model (Figure 7) in which nutrient zinc acts as a signal to induce the *cag* T4SS and promote *H. pylori*-dependent inflammation. After a neutrophil response develops through *cag* T4SS-driven IL-8 production or through induction of the Th17 response, deposition of CP sequesters zinc and manganese. The result of this sequestration is that the *cag* T4SS is switched off. This tightly controlled regulation may contribute to bacterial immune evasion and promote chronicity.

## Materials and Methods

### Ethics statement

All animal experiments were done in concordance with the Animal Welfare Act, U.S. federal law, and NIH guidelines. All experiments were carried out under an ACORP protocol approved by Vanderbilt University Institutional Animal Care and Use Committee (IACUC; V/10/410 and V/13/240) and the Department of Veteran's Affairs, a body that has been accredited by the Association of Assessment and Accreditation of Laboratory Animal Care (AAALAC). The human study protocol was approved by the Vanderbilt University and the Nashville Department of Veterans Affairs Institutional Review Board (#5190). Human subjects gave informed written consent.

### Bacterial strains, cell lines and culturing

*H. pylori* strains SS1 (a mouse-adapted clinical isolate), PMSS1 (the clinical isolate from which SS1 was derived), and the PMSS1 *cagE* isogenic mutant (PMSS1 *cagE::aphA*, a gift from M. Amieva) were used for these studies. PMSS1 was selected because it has a functional *cag* PAI and has the ability to colonize mice, and SS1 was selected due to its ability to colonize mice, although it lacks a functional *cag* PAI [57]. For infection assays, *H. pylori* strains were cultured on tryptic soy agar plates supplemented with 5% sheep blood or in *Brucella* broth supplemented with 10% fetal bovine serum at 37°C in room air supplemented with 5% CO_2_. For bacterial growth assays, *H. pylori* were grown in 60% *Brucella* broth plus 40% calprotectin (CP) buffer [16] supplemented with 10% fetal bovine serum (FBS) alone or supplemented with 50 µM zinc chloride and 50 µM manganese chloride, with increasing concentrations of CP at 37°C in room air supplemented with 5% CO_2_. Bacterial growth was quantified at 4, 12 and 24 hours by spectrophotometric reading of OD_600_, and at both 4 and 24 hours bacteria were subjected to serial dilution and plating onto tryptic soy agar plates supplemented with 5% sheep blood for enumeration of viable bacterial cells (CFU/mL).

For *H. pylori*-AGS cell co-culture assays, bacteria were grown in 60% *Brucella* broth plus 40% CP buffer supplemented with 10% FBS alone or supplemented with 100 µM zinc chloride, in the presence of 200 µg/mL WT CP, or 200–1200 µg/ml of ΔS1, ΔS2, double site mutant (DS CP) CP mutants. *H. pylori* were also cultured in the presence of the synthetic zinc chelator, N,N,N′,N′-tetrakis (2-pyridylmethyl) ethylenediamine (TPEN) (Sigma Aldrich) at a concentration of 5 µM. Bacteria for AGS co-cultures were enumerated by using the OD_600_ and bacterial coefficient established for this *H. pylori* strain using our spectrophotometer (this coefficient was determined by plating to enumerated viable bacteria). Purification of WT and mutant CP proteins was performed as previously described [16]. Adherence assay methods are in the Materials and Methods S1 file.

AGS human gastric cells (ATCC) were cultured to 70% confluency in RPMI medium supplemented with 10% FBS, 2 mM L-glutamine, and 10 mM HEPES buffer at 37°C in room air plus 5% CO_2_.

### IL-8 ELISA

*H. pylori* strains grown in various conditions of zinc availability were co-cultured with AGS cells at a multiplicity of infection of 100∶1 (as determined by spectrophotometric reading at OD_600_) for 4 hours. Cellular supernatants were collected and IL-8 was measured using an anti-human IL-8 ELISA (R&D), as previously described [32]. Bacteria grown in the presence of 5 µM TPEN or CP protein (WT, ΔS1 CP mutant, ΔS2 CP mutant, DS CP mutant) at either 200 µg/mL or 600 µg/mL were compared to those grown in the medium alone or in the presence of chelator plus 100 µM zinc chloride.

### NF-kB reporter assay

Human gastric AGS cells were stably transfected with an NF-kB-luciferase reporter, as previously described [25]. Briefly, eukaryotic cells were grown to 70% confluency, bacteria were added at a multiplicity of infection of 20∶1 (as determined by spectrophotometric reading at OD_600_), and co-cultured for 4 hours. Supernatants were collected, cells were lysed and luciferase activity was measured with the Promega E4030 luciferase assay system (Promega, Madison, WI). As a positive control, *H. pylori* PMSS1 grown in medium alone was used and normalized for 100% luciferase activity.

### Immunoblotting for phospho-CagA and total CagA

Phospho-CagA was detected by immunoblot, as previously described [25]. For detection of phospho-CagA, AGS cells were co-cultured with bacteria, as described for the IL-8 ELISA assays. AGS cells were washed twice with RPMI medium containing 1 mM sodium orthovanadate and pelleted by centrifugation (15,000 RPM for 3 minutes). Pellets were lysed in NP-40 lysis buffer containing Completer Mini EDTA-free protease inhibitor (Roche, Indianapolis, IN) and 2 mM sodium orthovanadate. Proteins were separated by soluble fractionation using 7.5% SDS-PAGE and immunoblotting with an anti-phosphotyrosine antibody (anti-PY99, Santa Cruz) or anti-*H. pylori* antibody, a polyclonal rabbit anti-*H. pylori* antibody described in [58]). Immunoreactive bands were visualized by ECL following incubation of the blot with HRP-conjugated secondary antibody. Detection of CagA and *H. pylori* soluble proteins was performed by stripping the blot with Restore Buffer (Pierce), and reprobing with either anti-CagA or anti-*H. pylori* primary antibodies.

### Animals and experimental *H. pylori* infection

Permission to use male and female IL-17RA-/- mice for the establishment of a breeding colony was obtained from Amgen (Seattle, WA). S100A9-/- mice were a gift from Wolfgang Nacken (Institute of Experimental Dermatology, University of Münster, 48149 Münster, Germany) to E.P.S. The generation of these mice was previously described [59]. CP-deficient mice lack the S100A9 component of the heterodimer and exhibit destabilization of S100A8 protein as well [60], resulting in deficiencies in metal sequestration [14]. Amgen's IL-17RA-/- mouse breeding colony is maintained at Taconic Farms. Helicobacter-free male IL-17RA-/-, S100A9-/- and WT mice (all C57BL/6 background; 8–10 weeks old, were used in all experiments). Mice were orogastrically infected with 5×10^8^ CFU *H. pylori* in 0.5 mL of *Brucella* broth twice over a 2 day period.

### Processing of mouse stomachs

At different time points post-infection mice were sacrificed by carbon dioxide inhalation and the glandular stomach was removed for analyses. The stomach was removed from each mouse by excising between the esophagus and the duodenum. The forestomach (nonglandular portion) was removed from the glandular stomach and discarded. The stomach was rinsed gently with PBS to remove food and cut into three longitudinal strips spanning both the antrum and corpus, which were used for quantitative bacterial culture, RNA extraction/real-time RT-PCR analyses, and histological examination. For culturing of *H. pylori* from the stomach, gastric tissue was placed into *Brucella* broth-10% FBS for immediate processing. For RNA extraction, the stomach was placed in RNAlater solution (Ambion) before being processed. A longitudinal strip from the greater curvature of the stomach was excised and placed in 10% normal buffered formalin for 24 hours, embedded in paraffin and processed routinely for hematoxylin and eosin (H&E) staining. Indices of inflammation and injury were scored by a single pathologist (M.B.P.) who was blinded to the identity of the mice. Acute and chronic inflammation in the gastric antrum and corpus were graded on a 0–3 scale. Acute inflammation was graded based on density of neutrophils and chronic inflammation was graded based on the density of lamina propria mononuclear cell infiltration independent of lymphoid follicles. Total inflammation was calculated as a sum of acute and chronic inflammation in the corpus and the antrum allowing for quantification of total inflammation on a scale of 0–12 [61]. Immunohistochemistry was performed using commercially available polyclonal rabbit anti-S100A9 antibody (Cat # NB110-89726, Novus Biologicals, Littleton, CO).

For quantitative culture, the gastric tissue was homogenized in *Brucella* broth using a tissue tearor (BioSpec Products, Inc. Bartlesville, OK). Serial dilutions of the homogenate were plated on trypticase soy agar plates containing 5% sheep blood, 10 µg/ml nalidixic acid, 100 µg/ml vancomycin, 2 µg/ml amphotericin, and 200 µg/ml bacitracin. After 5 to 7 days of culture under microaerobic conditions generated by CampyPak Plus Gas Pak system, *H. pylori* colonies were counted. Flow cytometry methods are in the Materials and Methods S1 file.

Because our study comparing PMSS1 and PMSS1 *cagE* mutant was designed to elucidate the regulation of the *cag* T4SS in response to a neutrophil-associated antimicrobial protein, the six week post-infection time point was an appropriate timepoint when neutrophils would be present [29] and the *cag* T4SS would still be functional [25], [57], [62].

### RNA isolation and real-time RTPCR

RNA was isolated from the stomach using the TRIZOL isolation protocol (Invitrogen, Carlsbad, CA) with slight modifications, as previously described [61]. RNA was reverse transcribed using the High Capacity cDNA Reverse Transcription Kit (Applied Biosystems, Foster City, CA). For real time RT-PCR, we used the relative gene expression method [63]. Glyceraldehyde 3-phosphate dehydrogenase (GAPDH) served as the normalizer, and tissue from a 5 uninfected WT mouse stomachs (or 5 uninfected human biospies) served as the pooled calibrator sample. All real time RT-PCR was performed using an Applied Biosystems StepOne Plus real time PCR instrument. Levels of cytokine expression are indicated as “relative units”, based on comparison of tissue from *H. pylori*-infected mice with tissue from uninfected mice (calibrator tissue) [63]. Primer and probe sets were purchased as Taqman Gene Expression Assays from Applied Biosystems (as pre-designed assays the annealing temperatures and amplicon length are available on their website). Primer and probe sets for eukaryotic genes were purchased as Taqman Gene Expression Assays from Applied Biosystems [S100A8 (Mm01220132_g1), S100A9 (Mm00656925_m1), GAPDH (Mm99999915_g1), human S100A8 (Hs00374264_g1), human S100A9 (Hs00610058_m1), and human GAPDH (Hs99999905_m1)].

### Field emission gun-scanning electron microscopy of *cag* T4SS pili

*H. pylori cag* T4SS pili were imaged by field emission gun-scanning electron microscopy (FEG-SEM) analysis using methods previously described [32]. Briefly, *H. pylori* cells grown under various conditions of zinc availability were co-cultured at a multiplicity of infection of 50∶1 with AGS human gastric epithelial cells on poly-L-lysine-treated coverslips (BD Biosciences) for 4 h at 37°C in the presence of 5% CO_2_. Cells were fixed with 2.0% paraformaldehyde, 2.5% glutaraldehyde in 0.05 M sodium cacodylic acid buffer for 1 h at room temperature. Samples were washed three times with cacodylic acid buffer before secondary fixation with 1% osmium tetroxide. Cells were subjected to sequential dehydration with increasing concentrations of ethanol before being dried at the critical point, mounted onto SEM stubs, painted with colloidal silver at the sample edge, and sputtered with 20 nm of gold-palladium coating. Samples were visualized with an FEI Quanta 250 FEG-SEM at high vacuum and micrographs were analyzed with Image J software.

### Statistical analysis

Statistical analysis of bacterial burden was performed after log transformation using unpaired two-tailed Student's *t*-test. Statistical analyses of IL-8 secretion, pilus quantification, expression data and luciferase activity were performed using paired two-tailed Student's *t*-test. Statistical analyses of histological inflammation scores were performed using Mann-Whitney U analysis. All data were derived from at least three separate biological replicates unless specified otherwise. Statistical analyses were performed using GraphPad Prism Software.

## Supporting Information

Figure S1 **Co-culture of** ***H. pylori*** **with CP or TPEN reduced bacterial viability.** A) WT *H. pylori* were cultured for 24 hours in medium alone or medium supplemented with 50 µM zinc chloride plus 50 µM manganese chloride (Medium+Zinc+Manganese) and with increasing concentrations of CP. B) WT *H. pylori* were cultured for 24 hours in medium alone or medium supplemented with 100 µM zinc chloride (Medium+Zinc) and with increasing concentrations of TPEN, a synthetic zinc chelator. Bacterial growth was evaluated by serial dilution and plating onto bacteriological media and determining the CFU/mL. *p<0.05, Student's *t* test, n = 3 biological replicates.
(TIF)

Figure S2 **Inflammation in WT and CP-deficient mice infected with** ***H. pylori*** **.** A) At 6 weeks post infection, levels of gastric inflammation trended higher in A9-/- mice compared to WT mice in groups infected with the WT *H. pylori* PMSS1 strain (*p* = 0.11), but not in groups infected with the isogenic PMSS1 *cagE* mutant (*p* = 0.3273). Data represent the mean +/− SEM per each group (n = 8–9 animals per group). Statistical analyses were performed for inflammation scores using Mann Whitney U test. B) Flow cytometry on mouse stomachs at 6 week post-infection was performed to quantify neutrophils (Gr1+CD11b+ cells) and C) macrophages, (CD11b+Gr1− cells). Statistical analyses were performed using Anova followed by an unpaired student *t*-test. n = 3–5 biological replicates.
(TIF)

Figure S3 **Adherence of** ***H. pylori*** **to AGS human gastric epithelial cells is unaffected by 4-hour pretreatment with CP or TPEN.** Bacteria were grown in medium alone or medium supplemented with the synthetic zinc chelator TPEN (TPEN), wild-type CP (CP) at 200 µg/mL alone or in the presence of 100 µM zinc chloride (+Zinc) for 4 hours prior to co-culture with AGS cells. Adherent bacteria were quantified by serial dilution and plating to determine CFU/10^5^ AGS cells. No significant difference in adherence to epithelial cells was detected. Bars represent the mean +/− SEM per each group (n = 3 biological replicates).
(TIF)

Materials and Methods S1 Materials and methods for the bacterial adherence assay and for flow cytometry are included.
(PDF)

Table S1 **Inhibition of H. pylori growth by CP at 48 hours.** The ability of CP and metal binding site mutants of CP to inhibit the growth of H. pylori in vitro was measured after 48 hours of culture with or without CP, as described in the materials and methods. Asterisks indicate *p<0.01, student t test compared to no CP condition. n = 3 biological replicates.
(PDF)

Table S2 **Realtime rtPCR for** ***s100a8 and s100a9*** **expression at 6 weeks post infection.** Relative Units of *s100a8* and *s100a9* transcript levels in WT mice infected with PMSS1 or PMSS1 *cagE* mutant (relative to an uninfected pooled sample from 4 mice).
(PDF)
