# Supplementary figures and images for: The Host Protein Calprotectin Modulates the Helicobacter pylori cag Type IV Secretion System via Zinc Sequestration
Source: PLoS Pathog. 2014 Oct 16;10(10):e1004450. doi: 10.1371/journal.ppat.1004450 (PMC4199781; doi:10.1371/journal.ppat.1004450)

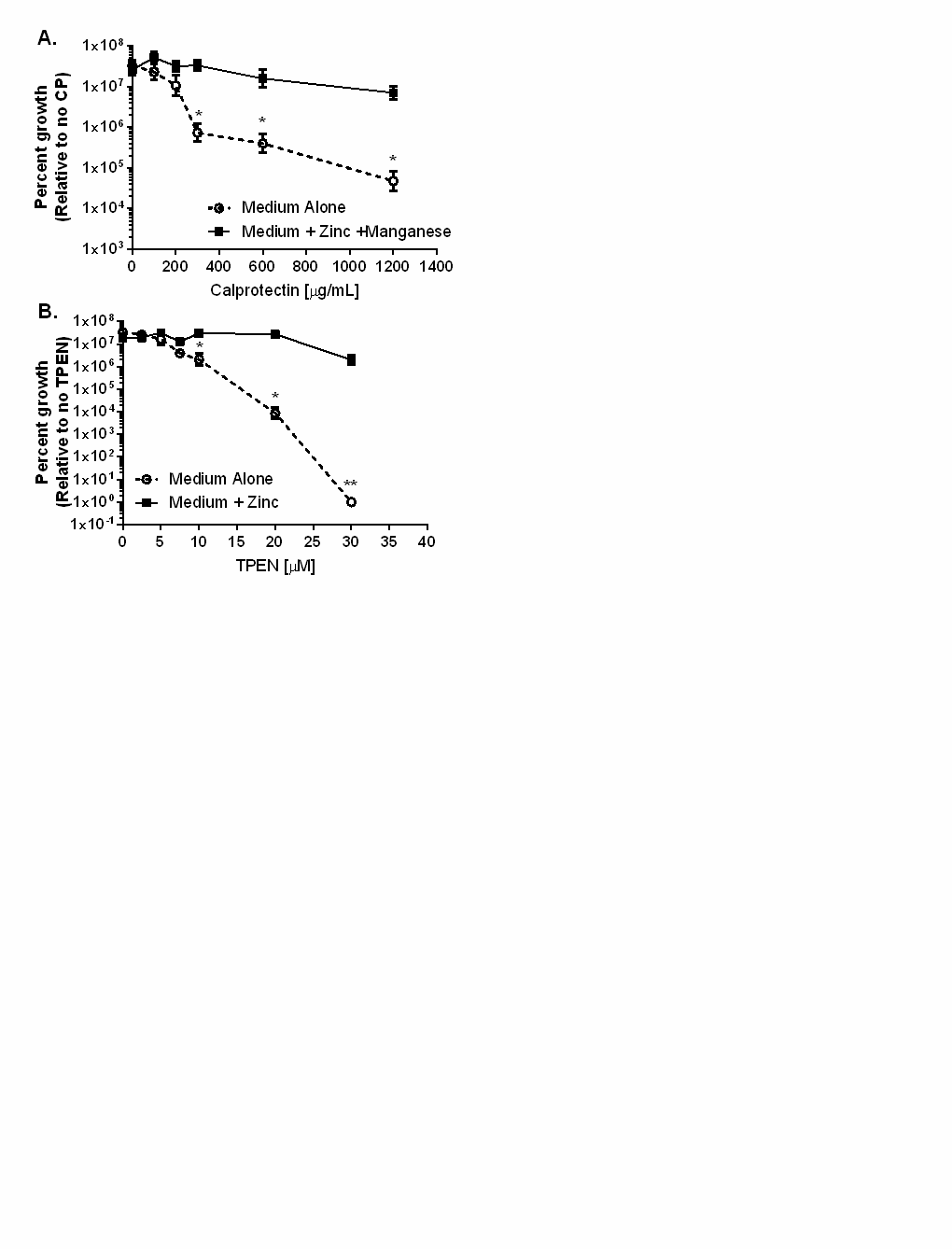

Supplement: Figure S1 — Co-culture of H. pylori with CP or TPEN reduced bacterial viability. A) WT H. pylori were cultured for 24 hours in medium alone or medium supplemented with 50 µM zinc chloride plus 50 µM manganese chloride (Medium+Zinc+Manganese) and with increasing concentrations of CP. B) WT H. pylori were cultured for 24 hours in medium alone or medium supplemented with 100 µM zinc chloride (Medium+Zinc) and with increasing concentrations of TPEN, a synthetic zinc chelator. Bacterial growth was evaluated by serial dilution and plating onto bacteriological media and determining the CFU/mL. *p<0.05, Student's t test, n = 3 biological replicates. (TIF) [file ppat.1004450.s001.tif]

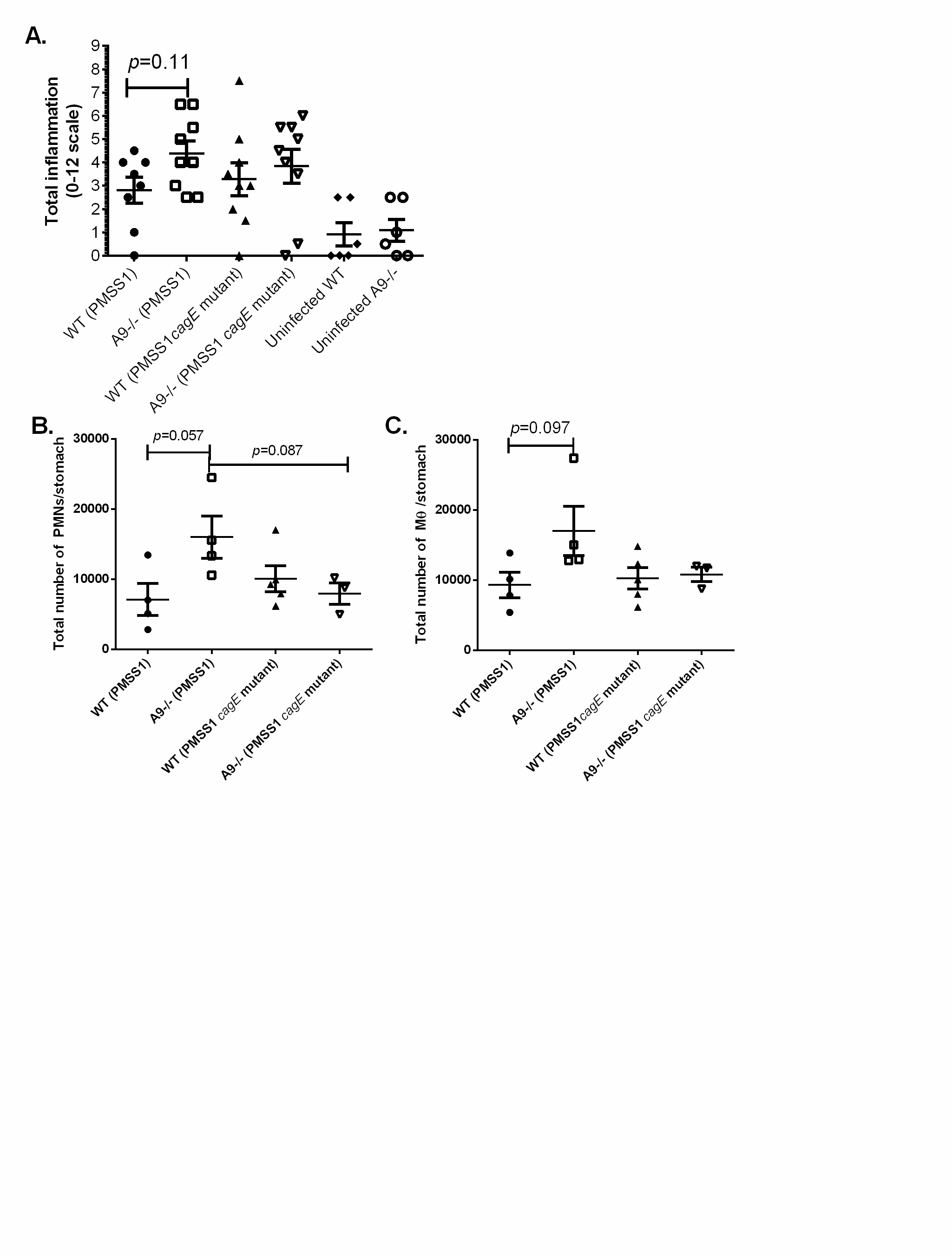

Supplement: Figure S2 — Inflammation in WT and CP-deficient mice infected with H. pylori . A) At 6 weeks post infection, levels of gastric inflammation trended higher in A9-/- mice compared to WT mice in groups infected with the WT H. pylori PMSS1 strain (p = 0.11), but not in groups infected with the isogenic PMSS1 cagE mutant (p = 0.3273). Data represent the mean +/− SEM per each group (n = 8–9 animals per group). Statistical analyses were performed for inflammation scores using Mann Whitney U test. B) Flow cytometry on mouse stomachs at 6 week post-infection was performed to quantify neutrophils (Gr1+CD11b+ cells) and C) macrophages, (CD11b+Gr1− cells). Statistical analyses were performed using Anova followed by an unpaired student t-test. n = 3–5 biological replicates. (TIF) [file ppat.1004450.s002.tif]

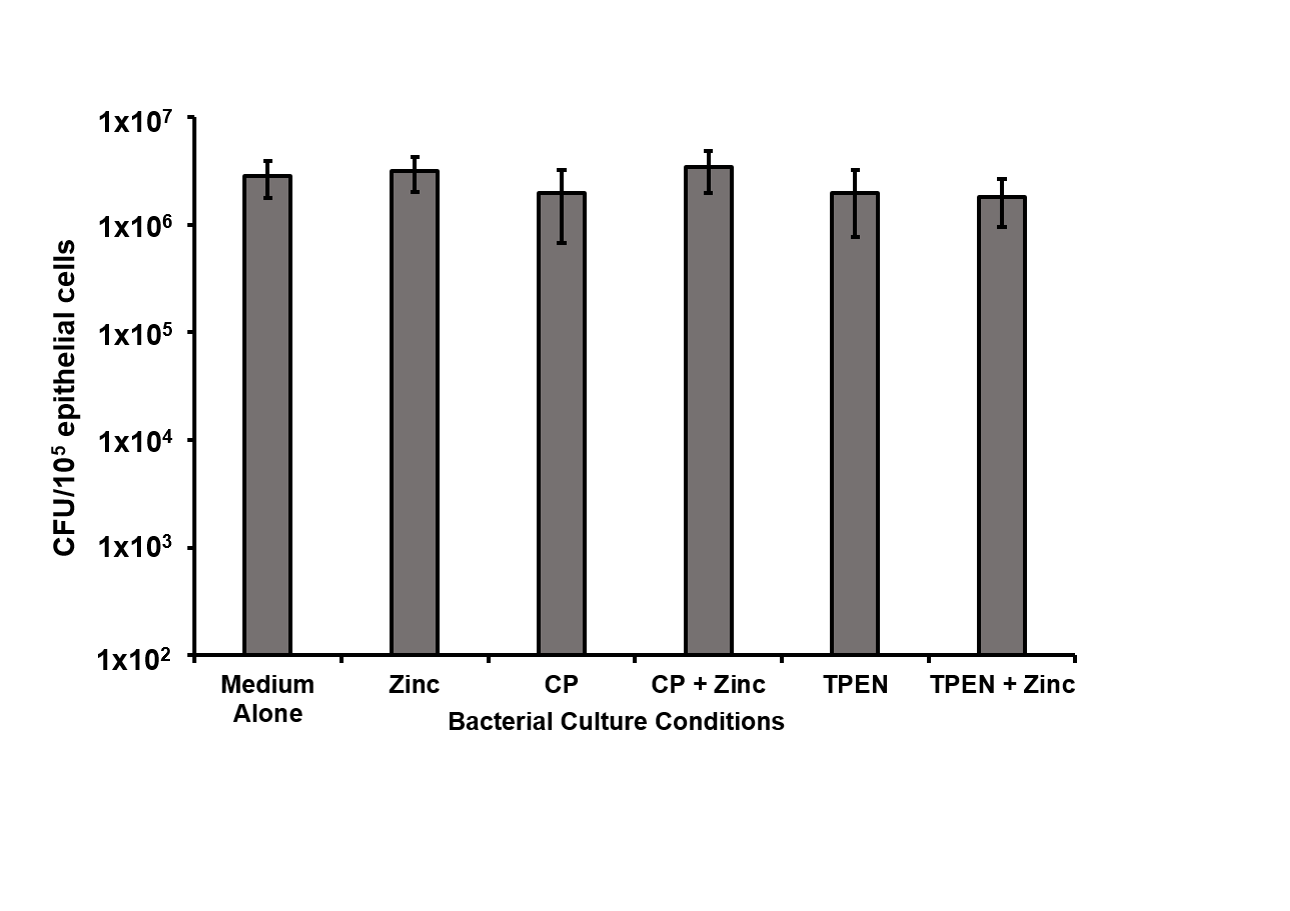

Supplement: Figure S3 — Adherence of H. pylori to AGS human gastric epithelial cells is unaffected by 4-hour pretreatment with CP or TPEN. Bacteria were grown in medium alone or medium supplemented with the synthetic zinc chelator TPEN (TPEN), wild-type CP (CP) at 200 µg/mL alone or in the presence of 100 µM zinc chloride (+Zinc) for 4 hours prior to co-culture with AGS cells. Adherent bacteria were quantified by serial dilution and plating to determine CFU/105 AGS cells. No significant difference in adherence to epithelial cells was detected. Bars represent the mean +/− SEM per each group (n = 3 biological replicates). (TIF) [file ppat.1004450.s003.tif]
