# Supplementary material for: The Host Protein Calprotectin Modulates the Helicobacter pylori cag Type IV Secretion System via Zinc Sequestration
Source: PLoS Pathog. 2014 Oct 16;10(10):e1004450. doi: 10.1371/journal.ppat.1004450 (PMC4199781; doi:10.1371/journal.ppat.1004450)
Supplement: Materials and Methods S1 — Materials and methods for the bacterial adherence assay and for flow cytometry are included. (PDF) [file ppat.1004450.s004.pdf]

Materials and Methods S1

Adherence Assays

To evaluate bacterial adherence to AGS gastric epithelial cells, bacteria were grown and co-cultured as described for FEG-SEM analyses (main text). After 4 hours of co-culture, the samples were washed three times with sterile phosphate buffered saline (PBS) and scraped to gather the biomass into a microcentrifuge tube. Serial dilutions were performed and samples were plated onto TSA plates supplemented with 5% sheep blood for enumeration of viable bacterial cells associated with AGS cells (CFU/10^5 AGS cells).

Flow cytometric analysis.

To analyze gastric cellular infiltrates, whole mouse glandular stomachs were harvested and processed using the Gentle Dissociator (Miltenyi Biotec). In short, the glandular stomach was rinsed in cold HBSS and then cut into 5mm pieces. The tissue pieces were transferred to a C-tube (Miltenyi Biotec, San Francisco, CA) with 5 ml of RPMI/10% FBS. The program m_imptumor 02 (37s) was run and dispase (final concentration of 0.32 mg/mL, Roche) and Collagenase D (final concentration of 0.30 mg/mL, Roche) was added to each tube. The tissue was incubated in the C-tube for 30 minutes at 37°C/5% CO2 while shaking. DNase (500 U/mL final concentration, Sigma) was added to each tube and the Gentle Dissociator was run on each sample twice (program m_imptumor 02). Dissociated tissue was gently vortexed, poured over a 70 μm cell strainer, and counted. The cells were spun down and washed and the gastric cells were stained with anti-Gr1 and anti-CD11b (BD Biosciences, San Jose, CA) as previously described [64]. Samples were collected and analyzed on a BD LSR II flow cytometer (BD Biosciences).
