# Supplementary material for: The Host Protein Calprotectin Modulates the Helicobacter pylori cag Type IV Secretion System via Zinc Sequestration
Source: PLoS Pathog. 2014 Oct 16;10(10):e1004450. doi: 10.1371/journal.ppat.1004450 (PMC4199781; doi:10.1371/journal.ppat.1004450)
Supplement: Table S1 — Inhibition of H. pylori growth by CP at 48 hours. The ability of CP and metal binding site mutants of CP to inhibit the growth of H. pylori in vitro was measured after 48 hours of culture with or without CP, as described in the materials and methods. Asterisks indicate *p<0.01, student t test compared to no CP condition. n = 3 biological replicates. (PDF) [file ppat.1004450.s005.pdf]

Table S1. Inhibition of *H. pylori* growth by CP at 48 hours. The ability of CP and metal binding site mutants of CP to inhibit the growth of *H. pylori in vitro* was measured after 48 hours of culture with or without CP, as described in the materials and methods. Asterisks indicate *$p<0.01$, student t test compared to no CP condition. n=3 biological replicates.

Calprotectin [concentration]	% Growth relative to no CP (+/-SD)
**CP [300 μg/ml]**	*35% (+/-7%)
**S1 mutant [600 μg/ml]**	85% (+/-4%)
**S1 mutant [1200 μg/ml]**	*58% (+/-11%)
**S2 mutant [600 μg/ml]**	94% (+/-12%)
**S2 mutant [1200 μg/ml]**	*60% (+/-5%)
**DS mutant [1200 μg/ml]**	86% (+/-3%)
**None**	100% (+/-3%)
