# Supplementary material for: The Host Protein Calprotectin Modulates the Helicobacter pylori cag Type IV Secretion System via Zinc Sequestration
Source: PLoS Pathog. 2014 Oct 16;10(10):e1004450. doi: 10.1371/journal.ppat.1004450 (PMC4199781; doi:10.1371/journal.ppat.1004450)
Supplement: Table S2 — Realtime rtPCR for s100a8 and s100a9 expression at 6 weeks post infection. Relative Units of s100a8 and s100a9 transcript levels in WT mice infected with PMSS1 or PMSS1 cagE mutant (relative to an uninfected pooled sample from 4 mice). (PDF) [file ppat.1004450.s006.pdf]

Table S2. Realtime rtPCR for *s100a8 and s100a9* expression at 6 weeks post infection. Relative Units of *s100a8* and *s100a9* transcript levels in WT mice infected with PMSS1 or PMSS1 *cagE* mutant (relative to an uninfected pooled sample from 4 mice).

***H. pylori* strain used for infection of WT mice**	**Relative Units of *s100a8*** (±SEM)	**Relative Units of *s100a9*** (±SEM)
PMSS1	1.18 (±0.07)	1.65 (±0.11)
PMSS1 *cagE* mutant	1.33(±0.10)	2.15 (±0.18)
